# Dynamics and Mechanical Stability of the Developing Dorsoventral Organizer of the Wing Imaginal Disc

**DOI:** 10.1371/journal.pcbi.1002153

**Published:** 2011-09-29

**Authors:** Oriol Canela-Xandri, Francesc Sagués, Jaume Casademunt, Javier Buceta

**Affiliations:** 1 Computer Simulation and Modeling (Co.S.Mo.) Lab, Parc Científic de Barcelona, Barcelona, Spain; 2 Departament d'Estructura i Constituents de la Matèria, Universitat de Barcelona, Barcelona, Spain; 3 Departament de Química-Física, Universitat de Barcelona, Barcelona, Spain; Ecole Normale Supérieure, France

## Abstract

Shaping the primordia during development relies on forces and mechanisms able to control cell segregation. In the imaginal discs of Drosophila the cellular populations that will give rise to the dorsal and ventral parts on the wing blade are segregated and do not intermingle. A cellular population that becomes specified by the boundary of the dorsal and ventral cellular domains, the so-called organizer, controls this process. In this paper we study the dynamics and stability of the dorsal-ventral organizer of the wing imaginal disc of Drosophila as cell proliferation advances. Our approach is based on a vertex model to perform in silico experiments that are fully dynamical and take into account the available experimental data such as: cell packing properties, orientation of the cellular divisions, response upon membrane ablation, and robustness to mechanical perturbations induced by fast growing clones. Our results shed light on the complex interplay between the cytoskeleton mechanics, the cell cycle, the cell growth, and the cellular interactions in order to shape the dorsal-ventral organizer as a robust source of positional information and a lineage controller. Specifically, we elucidate the necessary and sufficient ingredients that enforce its functionality: distinctive mechanical properties, including increased tension, longer cell cycle duration, and a cleavage criterion that satisfies the Hertwig rule. Our results provide novel insights into the developmental mechanisms that drive the dynamics of the DV organizer and set a definition of the so-called Notch fence model in quantitative terms.

## Author Summary

During development, tissues are shaped in order to form organs with specific functionalities. This process relies on mechanisms that control cell segregation and migration. These concepts are beautifully illustrated by the morphogenetic events that the imaginal discs of Drosophila undergo during metamorphosis. In particular, the cellular populations that will give rise to the dorsal (D) and ventral (V) parts on the wing blade are segregated and do not intermingle. The so-called organizer, a cellular population that becomes specified by the boundary of the D and V cellular domains, is responsible for this. Yet, how does the DV organizer robustly deal with the cellular growth in order to prevent cell mixing? Moreover, how can the organizer be conveniently scaled as the tissue grows? Herein we address these questions using a computational approach that takes into account the available experimental data. Thus, our study unveils the elements that are necessary and sufficient for understanding in a quantitative and predictive manner the dynamics, structure, and stability of a robust growing DV organizer: distinctive mechanical properties of cells, differences in cell cycle duration, and a well-defined cleavage criterion.

## Introduction

Patterning processes in multicellular organisms rely on faithful mechanisms of cell segregation and segmentation [1], [2]. These ideas are beautifully illustrated by the morphogenetic events that the imaginal discs of Drosophila undergo during metamorphosis [3]–[7]. There, the combined action of heritable selector genes confers location identities at the single cell level [8], [9]. For example, in the wing imaginal disc, *engrailed* and *apterous* genes endow cells with a posterior and a dorsal character respectively. Moreover, these genes grant some properties that determine cellular interactions that in turn restrict their locations, e.g. affinity and adhesion [2], [10]–[12]. Thus, cells under control of selector genes cannot intermingle freely and their positions become restricted to regions within the primordium: the so-called compartments [10], [11], [13]–[15]. The concept of compartment implies the existence of non-trivial boundaries that control cell migration [16]–[19]. While these lineage frontiers are not necessarily associated with morphological hallmarks of the organism, they play in all cases an additional and all-important role for setting the developmental plan. Such task is first driven by the differential gene expression pattern at both sides of the compartments interface, i.e. selector gene activity on versus off, that induces short range signaling between cells and promotes further patterning [8], [9], [14], [17], [19]. In particular, a cellular population becomes specified by the boundary defining the so-called organizer. Subsequently, signaling by morphogens towards the compartments takes place [3], [14], [20]. As a result, cells at the compartment bulk “read” the generated morphogen concentration gradient and obtain positional information [3], [21]–[25]. Therefore, an organizer acts in practice as the coordinate axis of a reference system. To this end an organizer must display some key features to guarantee its reliability as a source of positional information: the width of this cell population is constricted to few (two, three) cells [26], [27] and they develop maintaining a straight shape [28], [29]. Altogether, these findings meant a major breakthrough in modern Developmental Biology because of its powerful conceptual implications in terms of the modular design of multicellular organisms, conserved in both vertebrates and invertebrates, and its genetic foundation.

Since the discovery of developmental compartments, almost forty years ago, much progress has been attained with regard to the processes that lead to their formation and function [8]–[10], [26], [28]–[32]. Our recent contributions include the reverse engineering of the gene regulatory network that is responsible for the robust and stable patterning of the dorsal-ventral (DV) organizer and the elucidation of its minimal underlying network motif, the so-called *spatial toggle switch* [27], [33]. Thus, the underlying mechanisms that set the expression and activity pattern of the DV organizer and neighboring cells are now mostly clear from the point of view of a static tissue. Yet, many aspects of the functioning of the DV organizer of the wing imaginal disc still remain puzzling. In particular, how this pattern can be progressively and robustly scaled as cell proliferation advances remains a conundrum. Addressing such complex problem in an effective manner requires to transcend the molecular level and focus on its effects in terms of the mechanical interaction between cells. From that perspective, herein we propose an in silico framework that sheds light into the dynamics that shape the DV organizer for being an effective source of positional information and a cellular lineage controller. Based on the available experimental data, our approach falls into the field of Modeling and Computational Biology and introduces a realistic and novel description of the cellular dynamics of the DV organizer and neighboring compartments, leading to a series of quantitative predictions that can be experimentally tested.

**Figure 1:**
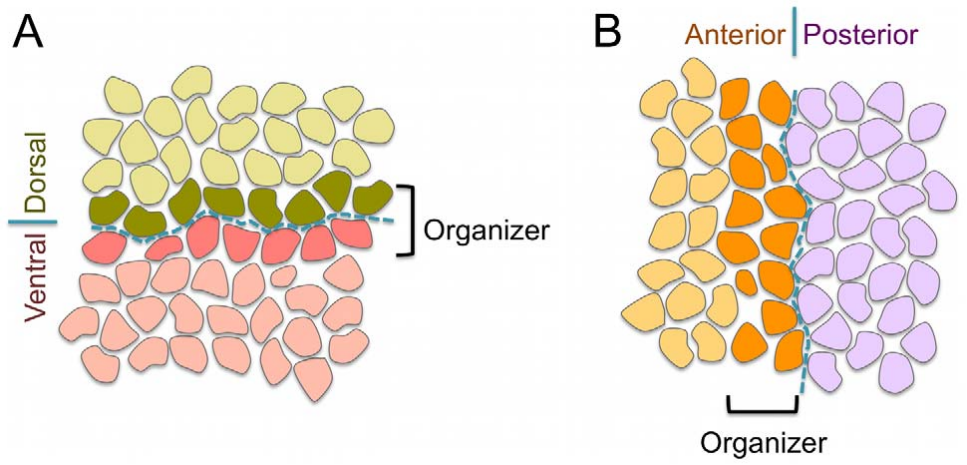
Schematic representation of the DV and AP organizers and boundaries. **A:** The DV organizer develops symmetrically with respect to the D and V compartments and acting as a fence prevents cells at the compartments bulk from mixing. The width of the DV organizer population is typically restricted to two cells. The DV boundary (blue dashed line) develops at the middle of the DV organizer and confer distinct mechanical properties to cells. **B:** The AP organizer is located at the anterior compartment. Importantly, the task of maintaining cells from opposite compartments separated relies on the boundary (blue dashed line) in this case (see text).

Within the aforementioned general developmental script about the formation and function of boundaries and organizers, some relevant peculiarities depend on the problem under consideration. Thus, in the case of the wing imaginal disc, the anterior-posterior (AP) organizer becomes established at the anterior side of the AP boundary, whereas the DV organizer is located at both sides of the boundary [17], [34], [35] (see Fig. 1). This has important implications with respect to the organizers characteristics and their role. In both cases the boundary is formed at the interface of two compartments. Still, the AP boundary strictly separates two cellular populations –the AP organizer, that belongs to the A compartment, and the P compartment– whereas the DV boundary develops embedded within the DV organizer population and the entity that keeps cells segregated from opposite compartments is the organizer itself rather than the boundary [17], [34], [35]. In other words, the separation between anterior and posterior cell populations is driven by an interface, namely the AP boundary, whereas the dorsal and vental cell populations are kept segregated by a cellular structure, namely the DV organizer. A fluid simile: in a two fluids mixture, the interface separating those would correspond to the AP boundary, whereas the DV organizer case would be comparable to a fluid film that intercalates between them. The mirror image nature of the DV organizer with respect to the compartments is due to a symmetric short signaling between compartments that sets the activation onset of the transmembrane receptor Notch [26], [27]. It has been suggested that such symmetry is necessary from a morphological point of view since the D and V compartments lead, upon development, to the specular dorsal and ventral surfaces of the wing blade in the adult organism. On the other hand, the DV organizer and neighboring cells give rise to the wing margin [16]. Other relevant differences between the AP and DV cases refer to necessary and sufficient conditions for their establishment. Whereas in the AP case the differential cell affinity between A and P cells, driven by the *engrailed* selector gene, seems to be required for lineage restriction, in the DV case the gene expression signature of the organizer (Notch activity) is necessary and sufficient for establishing a lineage barrier regardless of the identity of the cell populations [28], [29], [36], [37]. As a consequence, ectopic activation of Notch at either dorsal or ventral compartments recreates a functional organizer and, conversely, if Notch signal is blocked, then compartment cells can freely mix [36]. While there is an apparent contradiction in regards of the function of Notch for the maintenance of the DV organizer, transcriptional [38] versus non-transcriptional [28] –see also [31]–, it is clear that Notch receptor and its signaling pathway are indispensable elements for the establishment and maintenance of the DV organizer. Everything considered, researchers have adequately coined the term *Notch fence model* to describe these specific features [17].

Recent research has pointed out that mechanical effects play a central role in the function of the DV organizer. Thus, it has been shown that both F-actin and Myosin II accumulate by the zonula adherens at the junctions of the DV border [28], [29]. Running along the boundary, these components putatively promote cell adhesion and increase the cortical tension of cells. In agreement with these studies, it has been recently reported that actomyosin-based barriers are effective inhibitors of cell mixing in other developmental stages of Drosophila [32], [39]. These results provide evidence in favor of a crucial and active role of the cytoskeleton, and consequently of the mechanical effects, for keeping the straightness and fence-like features of the DV organizer. Importantly, it has been recently proved that, in addition to the differences in cell affinity, some of these contributions –increased cell tension– underlie the functioning of AP boundary too [39]. Moreover, other studies indicate that the consideration of dynamical and morphological factors related with the cell cycle is also required for understanding the stability and robustness of the DV organizer. During the course of development the increase in cell number of the wing imaginal disc is approximately 1000-fold. This poses the intriguing question of how the DV organizer deals with division events for maintaining its straightness, width, and stability. That is, how does the DV organizer pattern become robustly scaled as proliferation progresses? Related to that, it has been demonstrated in different contexts that the orientation of cell divisions determines the shape of developing tissues and organs [40]–[45]. In particular, it is now clear, either from measurements of the orientation of the mitotic spindle or of the post-mitotic cellular allocation, that cells of the DV organizer follow a division pattern that is different from cells at the bulk of the compartments, favoring the division plane to be perpendicular to the DV boundary [28],[41].

Nowadays it is widely recognized that in silico experiments are a powerful and effective tool for studying the dynamics of epithelial tissues like the wing imaginal disc [39], [45]–[52]. Following the seminal study of Weliky and Oster [53], the so-called vertex model was first introduced by Nagai and Honda [54]. The model exploits the polygonal-like morphology, the monolayer character, and the apicobasal mechanical polarization of epithelial cells to characterize them by a reduced set of points: the apical vertices. The dynamics of each cell vertex depends on the applied forces that derive from mechanical considerations, e.g. cytoskeleton activity. In the literature different examples are found where the vertex model has successfully described the wing imaginal disc. Recent advances include its packing, the AP compartmentalization [39], [50], the effects of the mechanical feedback on the tissue topology [52], and the alignment of the planar cell polarity domains with the proximal-distal axis of the wing [45]. However, to the best of our knowledge, no example has been reported so far where a realistic dynamics of the cell cycle, the cell growth, and the division events are also taken into account.

Within this framework our objectives are twofold. First, we propose an improved methodological approach for in silico experiments on epithelial tissue dynamics. To this aim, we present a simulation code based on the vertex model that includes the aforementioned dynamic and morphological effects in a realistic manner, i.e. cell cycle, growth, and cleavage criterion including stochastic variability, the anisotropic effects of the actomyosin cortical ring, and a boundary condition that does not impose an overall growth rate on the tissue. Second, and most important, we aim at elucidating the sufficient and required ingredients that endow the DV organizer with its features of functionality and robustness during the course of development as cell proliferation advances. To this end we test our model against the available experimental data and predict/quantify the effects when any of those components is missing. Our main conclusion is that the interplay between mechanical effects and the cell growth leads to the functionality and robustness of the growing DV organizer. Importantly, our results provide novel insights into the developmental mechanisms that drive the dynamics of the DV organizer and set a definition of the Notch fence model in quantitative terms and with regards to its sufficient and required contributions. Thus, we present evidence, both analytical and computational, that a distinctive regulation of the duration of the cell cycle is needed at the DV organizer for maintaining its features and stability, and that the cellular mechanical properties and the cleavage direction are coupled by the Hertwig rule. In addition, our in silico mutant analysis allow us to explore the role played by the differential affinity of cells at the compartments and the organizer and the actomyosin cable that develops at the DV boundary.

The paper is organized as follows. The Results section first introduces the wild-type situation and shows that our modeling reproduces the dynamics and structure of a stable DV organizer that agrees with the available experimental data in terms of topological/size distributions [50], cell division patterns [28], [41], cell response to ablation experiments [50], and geometry adopted by ectopic organizers [28], [29]. In addition, we also show that the DV organizer is robust with respect to mechanical perturbations like fast growing clones and parameter variation. Thus, our results unveil the mechanical and dynamical ingredients that are sufficient for explaining in a quantitative and predictive manner the growth of the DV organizer; in order to demonstrate that those are also necessary, we perform in silico experiments with lack-of-function mutants. In the Discussion section we elaborate the main conclusions that derive from our study and comment on their implications. Finally, in the Methods section, we flesh out our approach by describing the dynamical vertex model, and the implementation of the cell cycle and the cell division events. Therein we also detail the values of the parameters used in our simulations, the initial and boundary conditions, and the rules that control the cellular character.

## Results

### Wild-type Background: Sufficient Ingredients for Keeping the Structure and Stability of the Growing DV Organizer

**Figure 2:**
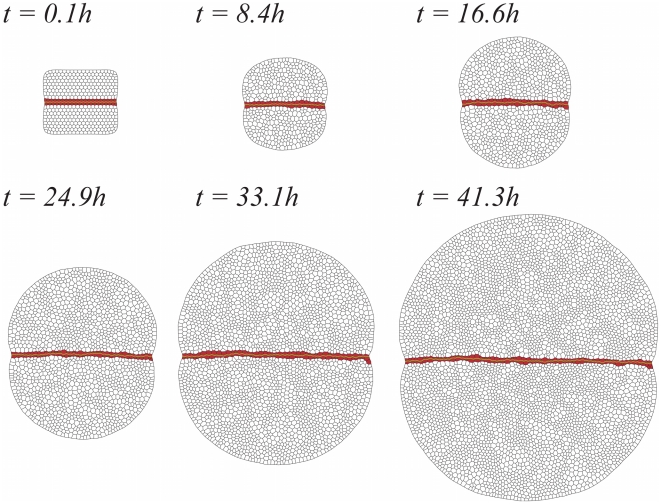
Snapshots of the developing in silico wild-type wing disc. As time evolves the DV organizer (red cells) grows straight maintaining a two-cells width and restricts cells from the compartments bulk (white cells) for mixing. The DV boundary has been highlighted in green.

Fig. 2 shows several snapshots that illustrate, from left to right and from top to bottom, the temporal evolution of a growing tissue under wild-type conditions (see also Video S1 in the Supporting Information). Herein the term wild-type indicates that, as shown below, with the parameters used in our in silico experiments (see Methods) we are able to reproduce both qualitatively and quantitatively the dynamics of the DV organizer of an in vivo wild-type experiment (see also robustness results below). The primordium comprises, as prescribed by the initial condition, two cell populations with differentiated mechanical properties: cells at the bulk of the compartments (white) and the DV organizer cell population (red). As specified in the Methods section, the character of organizer cells can be lost depending on whether or not the cellular environment is able to maintain their Notch activity. Both compartments are identical in size and properties, i.e. dorsal/ventral regions satisfy the same dynamics. In that figure, the DV boundary has been highlighted by simply connecting the cell membrane edges of the DV organizer at opposite compartments. The initial phase of the cell cycle at each cell is taken as random and uncorrelated with respect to that of its neighbors. Yet, during the evolution of the growing tissue, correlations between the cell cycles of neighboring cells (clustering) naturally develop due to the division process (see Video S2). Our results about cell clustering are in qualitative agreement with experimental results that have shown that dividing cells are found throughout the entire disc as single cells or clusters of 2–10 neighboring cells [55]. Still, Milán and coworkers demonstrated that clustering is also driven by cell signaling that help mitotic cells to recruit neighboring competent cells, a problem that we have disregarded. Moreover, recent experimental results have revealed that the amount of clustering strongly depends on the experimental protocol [52]. Everything considered, a thorough quantitative comparison with experiments is difficult.

Starting from the initial condition, the tissue is evolved 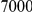 dimensionless time units (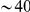 hours) up to reaching a population of 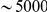 cells (i.e. each cell undergoes, on average, three cell cycles). As detailed in the Methods section, the simulation follows the dynamics as determined by Eq. (3), including simultaneous growth and division of cells.

**Figure 3:**
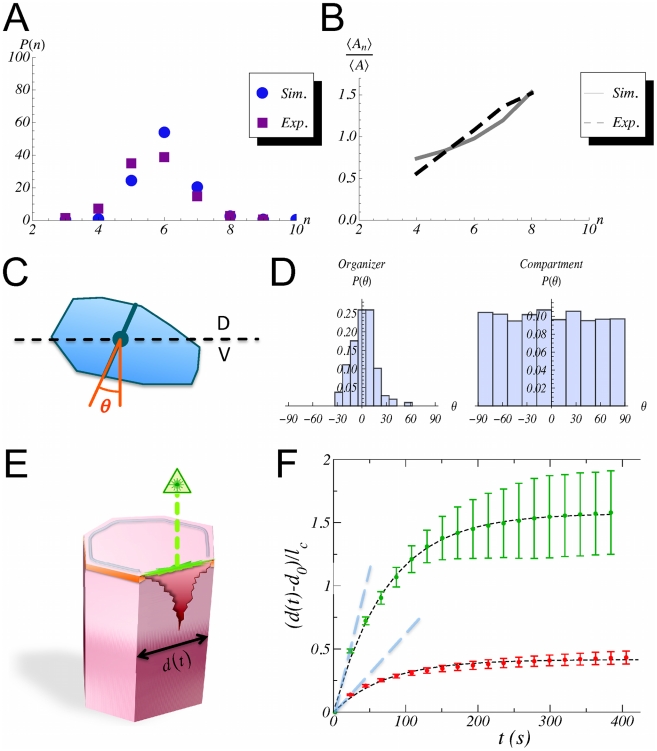
Quantification of the packing statistics and relaxational dynamics upon laser ablation. **A:** Comparison between the histogram of the number of cell sides in simulations and in vivo experiments (experimental data from ref. [50]). **B:** Comparison between the distribution of the normalized average area as a function of the number of cell sides in simulations and in vivo experiments (experimental data from ref. [50]). In both cases the overall trends and figures are in agreement. **C:** Schematic representation of the quantification of the cleavage orientation. By computing the principal axes by means of the inertia tensor (see text) we determine the elongation axis and divide the cell along an orthogonal direction to the latter (solid blue line). The cleavage direction statistics quantifies the angle 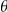 with respect to an off-lattice direction that defines the DV axis (black dashed line). **D:** Histogram of the cleavage directions in the organizer population (left) and the compartments bulk (right). The data reveals that the mechanical constraints force cells of the DV organizer to elongate along the DV axis. On the contrary, cells at the compartments bulk do not show any preferential direction for their divisions. **E:** Schematic representation of the laser ablation experiments. In our simulations when a cell edge is ablated we assume that the damage that is caused destroys the adherent junctions (orange band) and also the region of the cortical ring related to that edge (grey ring). Yet we suppose that the rest of the ring is functional (see text). **F:** Simulations results of 20 ablation experiments in cells of the compartments bulk (red circles) and in organizer cells when the ablated edge corresponds to the DV boundary (green circles). The error bars account for the standard variation over experiments. In both cases the relaxation time is 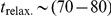 seconds as deduced by the fitting to an exponential decay (black dashed curve). The actomyosin cable increases the tension as revealed by the increased displacement between vertices when the ablated edge belongs to the DV boundary. Those differences in cell tension can be quantified by the initial velocity of the vertices expansion (slopes of blue dashed lines): 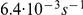 (compartments bulk) and 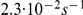 (DV boundary).

Visual inspection of Fig. 2 indicates that under these conditions the DV organizer grows straight (see quantification below) keeping a two-cell wide population and separating cells of opposite compartments. In addition, Fig. 2 reveals the robustness of the DV organizer scaling process as cell proliferation advances since it is able to cope with the stochastic variability of the cleavage orientation (see Methods). Further robustness analyses to test the stability of the DV organizer can be done by means of mechanical perturbations and parameters variation (see below). Besides these observations, different quantitative characteristics of the statistics of the growing tissue can also be extracted from the simulations in order to compare with experimental data. On the one hand, we analyze the cellular packing in relation to: i) the histogram of the number of cell sides (neighbors) and ii) the normalized average area distribution as a function of the number of cell sides. To this respect, we do not observe major differences when comparing cells at the organizer and at the bulk (data not shown). On the other hand, we compute the distribution corresponding to the cumulative statistics of cell division orientations. As expected, in this case we do need to distinguish the populations from the organizer and from the compartments bulk. Results of these analyses are summarized in Fig. 3.

With regard to the packing statistics data, Figs. 3A–3B show the comparison of our in silico experiments with in vivo data of growing third instar wing imaginal discs from other researchers [50]. Trends and figures are well-reproduced; in particular we recover the preference for hexagonal coordination. The agreement indicates a reasonable accurate choice of parameters. As a possible source of the discrepancy, we note that the experimental figures derive from the analysis of static images while ours are obtained from the cumulative statistics of the tissue dynamics at different times. Interestingly, the histograms for cell division orientation reveal how the mechanical properties contrain the cellular cleavage. The angle of division is measured using as a reference an axis perpendicular to the DV boundary such that a null angle corresponds to cells that cleave orthogonally to the DV boundary (see Fig. 3C). Fig. 3D reveals the differences between cells at the bulk (right) and cells at the DV organizer (left). We recall that besides the Hertwig rule (see Methods), no direction for the division is imposed. Consequently the histogram unveils a geometrical property of cells at the DV organizer: driven by the mechanical forces, those cells elongate along the DV boundary. Interestingly, this elongation is counterintuitively performed under the expense of positive line and cortical tensions (see parameters values below). This persistence of oriented division is a distinctive attribute of organizer cells that is lost when the bulk compartment population is analyzed similarly. In that case the distribution is almost uniform thus indicating the lack of any spatial anisotropy in the division direction. With regard to the statistics of cell division orientation, it is worth mentioning an apparent discrepancy with the data from Major and Irvine [28]. There, a much more scattered division-orientation distribution for cells at the DV organizer, more uniform, yet different, to the cells at the compartment bulk, is reported. This disagreement can be attributed to the fact that their statistics actually refers to the orientation of the mitotic spindles of each cell relative to the nearest DV interface which may fluctuate significantly with respect to the actual cleavage direction. In fact, with respect to cells that belong to the organizer, our data are in better agreement with studies performed by Baena-López and coworkers that also quantify the post-mitotic allocation [41]. Still, there is some discrepancy that can be ascribed to the fact that these authors analyze longer developmental times. As for cells in the compartments, our data agree better with those of Major and Irvine since their analysis, as ours, focuses on regions close to the organizer as opposed to the studies of Baena-López et al. that explore longer developmental times and larger regions of the wing disc that contribute to the wing blade elongation.

Further analysis of the model that allows for quantitative comparison with in vivo experiments addresses the cell response upon membrane ablation. Herein, we introduce these in silico experiments as a novelty. By checking the relaxation of the network of cell links, the implementation of these in silico experiments allow to establish the value of some parameters of the model. Unfortunately, there is a lack of systematic characterization of the damage that the laser wound induces on the cells cytoskeleton. The outcome of the experiments presents in fact high variability [39], [50]. In our case, the ablation is implemented by suppressing a cell edge and, as a consequence, we eliminate for the corresponding cells and vertices all the energetic terms that depend on the suppressed edge, except for the elastic contribution due to the area. In particular, we remove the adhesion (line tension) contribution and further assume that the cortical ring is well attached to the cell membrane and consequently the wound modifies its properties by just removing the contribution of the wounded edge to its perimeter, while keeping intact the functionality for the rest of the ring (Fig. 3E). The energy relaxation is then analyzed as in the in vivo experiments by monitoring the time-dependent separation of the two vertices whose common link has been removed, 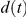, with respect to the equilibrium distance 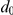. As shown in Fig. 3F, this relaxation is very close to exponential (see also Video S3), and thus characterized by a single time scale. Indeed the evolution is well fitted by the expression:
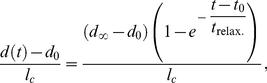
(1)


where 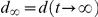, and 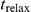 is the relaxation time scale. We perform in silico ablations of different cell edges: those that belong to the DV boundary, edges from the organizer that do not belong to the DV boundary, and edges from cells of the compartments bulk. In all cases a complete relaxation is achieved at times of the order of 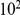 seconds. In particular we obtain 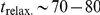 seconds. Moreover, edges from cells of the compartments bulk and edges from the organizer cells that do not belong to the DV boundary, behave dynamically in a similar manner (data not shown). The main differences arise when comparing the displacement between edges that belong or not to the DV boundary. In that case, and in agreement with in vivo experiments in the context of the AP boundary [39], [50], the former develops an increased displacement due to a larger tension. Those differences can be characterized by the initial velocity of the expansion: 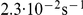 and 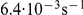 respectively, that is, a 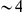 fold increase. We point out, that the comparison of our ablation data with those experiments in the context of the AP boundary is not feasible from a quantitative point of view since a different cellular environment is analyzed. Still, we expect our analysis to be predictive in that regard since an increased tension at the boundary has been experimentally reported in both cases.

**Figure 4:**
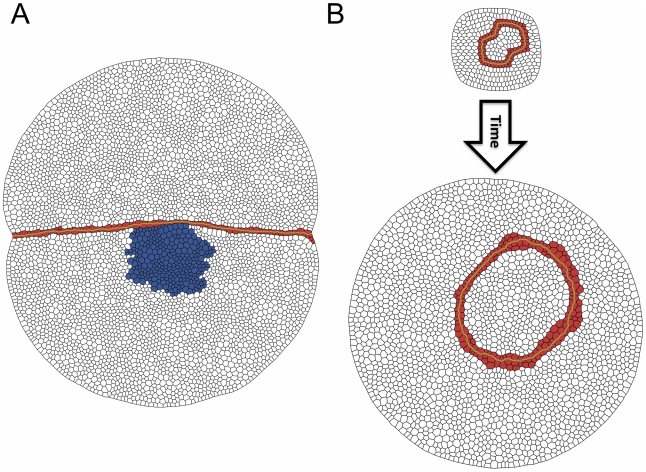
Perturbation experiments in a wild-type background. **A:** A clone of rapid replicating cells (blue) exerts a differential pressure over the DV organizer. Yet the latter maintains its stability over the course of growth. **B:** An ectopic organizer with a close shape develops minimizing its surface energy as seen in experiments.

**Figure 5:**
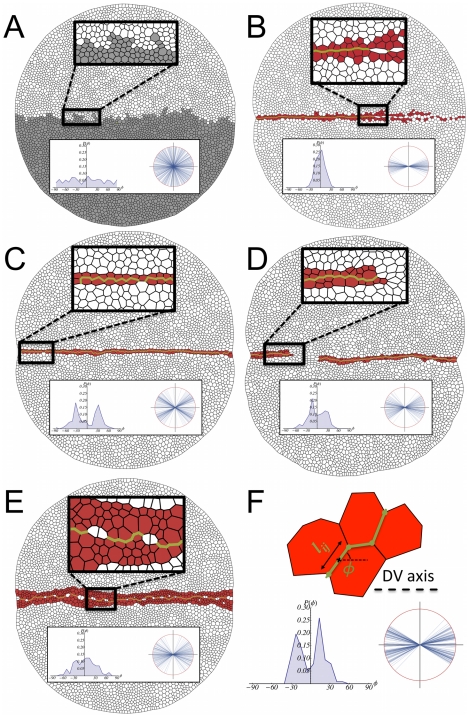
Mutant analysis. **A:** In the absence of a DV organizer, cells of opposite compartments intermix. **B:** The differential cell adhesion is removed in this simulation and as a result the organizer cannot maintain its stability. **C:** If the actomyosin cable is removed the width of the organizer is reduced at many locations to one cell and is not robust to perturbations. **D:** A criterion for cell cleavage is required for maintaining the stability of the boundary. If no rule is prescribed (here cells divide at random orientations) then the DV organizer easily breaks leading to cell intermingling between compartments. **E:** The duration of the cell cycle is set to be the same in the whole disc. This produces a wiggly boundary and a wide organizer. **F:** In order to quantify the structure of the boundary we evaluate the angle 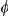 for all cell edges 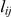 that define the boundary as illustrated on the top of this panel. On the bottom we show the results of the wild-type situation (Fig. 2) in regard to the representation of those angles in a polar plot (right) where all edges lengths are normalized to the unity and their associated histogram (left). The insets in panels A–E reveal that the mutants lead to boundaries/organizers that are weaker and/or wigglier than the wild-type (see text).

Additional analyses can be made in order to test that the components considered in our model are sufficient for reproducing and understanding the dynamics and growth of the DV organizer. In particular we focus on its stability against mechanical perturbations and on its dynamics when it is ectopically induced. The first test refers to the mosaic technique where clones of rapidly replicating cells are placed near the organizer (Fig. 4A). In our in silico experiments those cells have the same mechanical properties that cells at the bulk, yet their duplicating time is 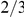 of that of the latter. As a consequence of its growth advantage, the clone rapidly extends and exerts pressure over the neighboring organizer. This force is revealed by the bending of the DV organizer around the location of the clone (see also Video S4). Still, in agreement with experimental results [56], the organizer grows intact and remains robust keeping the clone lineage restricted to its compartment. This fence-like picture contrasts with the situation where D and V compartment cells are placed in contact in the absence of an organizer (see Fig. 5A). In that case, the compartments populations progressively mix and start to interdigitate as cellular proliferation advances.

A second test has to do with another mosaic experiment. Clones at the compartment bulk driven by the ectopic expression of Delta in dorsal cells or that of Fringe in ventral cells are able to induce a functional organizer with fence-like properties as the wild-type one. Interestingly, the geometry of these clones evolves towards a roughly circular shape [28], [29]. We mimic those experiments in silico by ectopically placing an organizer in the compartment bulk. Its initial shape is chosen to be not circular but “eight”-shaped while the rest of its properties are the same that in the wild-type situation. As shown by our simulations (Fig. 4B, see also Video S5), the system evolves in agreement with the experimental observations rounding up the organizer region, very much like fluid interfacial systems would do in order to reduce the surface energy due to surface tension.

The biological plausibility of our proposal can be also evaluated from the perspective of the robustness with respect to parameters variation (see Methods). This analysis addresses also the degree of freedom for choosing the wild-type parameter set. In this regard, 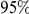 of the parameter sets that lie within a distance 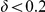 develop a functional DV organizer: it does not break within the temporal window of interest. Parameter sets that lie within a distance 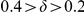 produce a functional organizer in 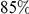 of the cases.

Additionally, we evaluate the effect of varying the external line tension. If the latter is decreased by a 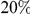, then the organizer develops robustly. On the other hand, if it is increased by a 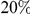, then some simulations reveal that it threatens to break at some points when subjected to perturbations. Still, the dynamics is in all cases similar to the wild-type: we do not observe significant changes in the growth rate and the organizer keeps the compartments segregated (see Fig. S2). Therefore, our analyses reveals a robust mechanism for maintaining the mechanical stability of the DV organizer.

### Mutant Analysis: Necessary Ingredients for Keeping the Structure and Stability of the Growing DV Organizer

In the previous section we have shown that our model includes ingredients that are sufficient to capture a repertoire of experimental observations involving both compartment cells and DV organizer cells in terms of the structure, stability, and dynamics of the latter. We now turn to check to what extent each of the components of the model is indeed required to account for the observed phenomenology. Our approach is based on different in silico experiments that mimic “lack-of-function” mutants by suppressing individual ingredients (one at a time). We point out that while some of those “mutants” are not easily feasible, and consequently difficult to test, e.g. randomizing the cleavage direction at the DV organizer population, they do provide crucial information for analyzing their importance.

The mutants considered are depicted in Fig. 5A–E, where a late snapshot of the growth after 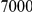 dimensionless time units (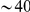 hours) is shown for comparison with the wild-type case of Fig. 2 (see also Videos S6–S10). While specific mutants reveal the role played by individual components, the best way to test the fence-like functionality of the DV organizer for restricting cell migration is to remove completely the organizer cells, e.g. a disc in the absence of Apterous activity [57]. Results in this direction are shown in Fig. 5A where it is evident that cells at opposite compartments intermix generating a finger-like pattern. Our first mutant test, Fig. 5B, refers to the case when the affinities (line tensions) between all cells, both bulk and organizer cells, are prescribed identical. Namely, organizer cells having the same affinity between them that cells at the bulk have, i.e. 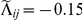 (see parameters values below). However, we retain the differential values for both the line tension and the contractility terms that account for the actomyosin cable at the DV boundary. Under these conditions the DV organizer is largely disrupted and the fence-like properties disappear. The opposite situation is represented in Fig. 5C where we maintain the heterogeneous affinities between organizer and bulk cells, but we eliminate the actomyosin cable by suppressing its distinctive tensile term. Although the DV organizer is not wiped out as severely as in the previous case, the integrity of the organizer is noticeably weakened (it lacks robustness), the DV boundary becoming less straight (see quantification below), and threatens to be ruptured at several points under prolonged proliferation (notice that at many locations the width of the organizer has been reduced to a single cell). This result is in agreement with experimental results showing that when the actomyosin cable is removed, the effectiveness of the compartment segregation is reduced [29].

The orientation of cell division is examined in Fig. 5D. This panel reproduces the result of the simulation when all cells in the tissue divide at random in terms of the cleavage orientation, i.e. cells do not follow the Hertwig rule. As expected from the results shown in Fig. 3D, this ingredient turns out to have a large impact on the stability of the organizer. Indeed, the DV organizer cannot maintain its stability and becomes easily disrupted. The role played by the distinctive cell cycle durations between organizer and bulk cells is finally analyzed in Fig. 5E. There, the differences in duration are removed and all cells are considered to have the same (mean) lifetime regardless of their lineage. In this case we observe that the DV boundary becomes very wiggly, and the DV organizer becomes wider. Note also that some cells cannot maintain Notch activity due to the widening.

The structure and straightness of the DV boundary can be characterized by measuring the angle 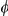 with respect to the DV axis for the cell edges 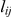 that define it, as shown in Fig. 5F. In our simulations, in order to discard artifacts in the quantification, we check that 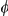 and 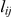 are not correlated quantities (data not shown). Our data reveal that the straightest boundary corresponds to the case where the same affinities apply to all cells (B). However, we recall that in this last case the DV boundary is easily disrupted and does not maintain its integrity in many regions. All other mutants lead to boundaries that are more wiggly than the wild-type case. Moreover, the histograms of angles indicate that in the wild-type situation the boundary preferentially organizes in a 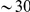 degrees zigzag configuration. The latter is also true for the cases C and D, yet showing a larger dispersion that contributes to their “waviness”. We notice that this 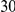-zigzag organization of the boundary is related with the predominant hexagonal topology of the tissue and not with the variability introduced in the cleavage orientation (as large as 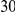 degrees, see Methods) as exposed by case D where the direction of division is random. The case where the organizer is removed, A, obviously displays the more outspread distribution with an almost uniform profile. In contrast, case B shows an uni-valuated distribution around zero degrees. Finally, case E reveals a wiggly organization of the boundary with an outspread angle distribution. Still, the latter is not uniform as in case A and indicates a preferential organization around 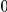 degrees.

Overall, we have shown that the distinctive mechanical properties of cells, the differences in cell cycle duration, and a cleavage criterion (Hertwig rule) are required elements for understanding the dynamics, structure, and stability of a robust growing DV organizer.

## Discussion

Herein we have proposed a dynamical vertex model to study the different dynamical ingredients that are both necessary and sufficient to understand, at a quantitative level, the mechanical maintenance of the DV organizer in the wing imaginal disc within the developmental time window we consider. Thus, we have shown that our model is able to reproduce, quantitatively when such data are available, the reported phenomenology in terms of packing statistics, division orientation, robustness against mechanical perturbations, relaxation dynamics upon membrane ablation, and the geometrical rearrangements of an ectopic organizer. Additionally, the validity of our model has been also corroborated from the viewpoint of the robustness with respect to parameters variation and to noise in the cleavage orientation. Importantly, we have presented evidence, both analytical and computational, that a distinctive regulation of the duration of the cell cycle is needed at the DV organizer for maintaining its features and that the feedback between the cellular mechanical properties and the cleavage orientation are coupled by means of the Hertwig rule. Whether or not these differences in cell cycle duration and the cleavage criterion are also necessary in the AP case is a subject of further research. Moreover, we have shown by means of an in silico mutant analysis, the effects of different contributions to the dynamics and regulation of this developmental structure and we have proposed a way to geometrically quantify the organization of the boundary.

Our approach does not take into account either the molecular effectors or the interactions and pathways that underlie the specific functionalities that we have reviewed herein. Instead, we make use of an alternative procedure of analysis in order to evaluate their consequences in an effective way. The computational approach is then particularly valuable to the extent that it allows the implementation of tests that may not be easily feasible in vivo. All in all, our study provides a definition of the Notch fence model in quantitative terms and provides the elements that are both sufficient and required to keep and scale a robust and stable organizer with a well defined width over the course of development.

There are crucial differences in our modeling approach with respect to previous implementations of the vertex model in the context of wing imaginal disc development [39], [45], [50], [52]. First, we have included extra energetic terms in order to account for the asymmetry of actin-myosin expression at different cell edges. Second, our model includes a realistic, stochastic, dynamics of the cell cycle duration, its relation to the cell area growth, and a implementation of the Hertwig rule with cell-to-cell variability. Third, our modeling allows for simultaneous cellular growth and a continuous time description that permits to conduct, among others, in silico ablation experiments that provide relevant biomechanical information. Finally, in contrast to other approaches that assume periodic boundary conditions our simulations implement free-boundary ones that do not need to impose a rate for tissue growth. There are certainly consequences derived from this simulation scheme, namely, the pinching of the organizer and the packing properties (e.g. orientation) of cells at the periphery. The external border is pinched due to the extra tension and pulling exerted by the actomyosin cable that runs along the DV boundary. In addition, since the line tension is larger at the periphery than in the compartments, the best energetic strategy for cells at the periphery is to minimize their surface of contact with that external border and, consequently, cells elongate perpendicularly to it. Still, those effects are arguably only local and not relevant for the dynamics and overall behavior of the tissue. The fact that cells grow simultaneously lead to changes with respect to the topological properties observed in simulations using a quasistatic approximation. In particular, if cells are growing concurrently the appearance of a transient synchronization of the cell cycles, i.e. temporal correlations, leads to an increase of the tissue local pressure that is able to modify the distribution of cell areas and the statistics of cell neighbors. Indeed, at first sight any snapshot of the cell packing reflects this dynamics by exhibiting two classes of cells, those that are latent, with a characteristic minimal size, and those that are growing, with larger sizes. This feature can often be visualized in real epithelia and its precise quantification in the particular context of the wing imaginal disc is now possible using the Fucci technique [58]. The simultaneous growth of many cells explains why the quantities measured in our simulations fit reasonably well the experimental observations, even though the mechanical parameters lie in different regions of the phase diagram as compared to the case of fitting experimental data in quasistatic models (see [50]). Incidentally, the values of the parameters reveal a complicated compromise, in some cases counterintuitive, between the energetic contributions.

As a matter of discussion, we comment on the rule we implemented for maintaining the genetic profile of organizer cells (see Methods). We effectively put into practice the fact that, during the temporal window of our interest, keeping Notch activity at organizer cells requires intercellular signaling between cells at the organizer and neighboring cells at the compartments via ligand-receptor binding [26], [27]. In this context we have also considered the role of filipodia for robust signaling as recently reported [59]. If the rule for maintenance is ignored and the genetic profile of cells is simply inherited following a division event, then the obtained results, both for the wild-type and the mutants, are totally equivalent. In fact, the only case where some small differences can be seen corresponds to the case where the cell cycle durations are the same (data not shown). This poses the interesting question about the relative importance of signaling versus mechanical and dynamical effects for the maintenance of the DV organizer during different developmental stages. At every developmental stage signaling is indeed fundamental. As a matter of fact, at early stages, mechanical and dynamical inputs do not seem to play a crucial role and signaling between compartments is the driving force for both, the formation and the stability, of the organizer [27]. Complementarily, our results suggest that, once the organizer has been established, the mechanical and dynamical contributions become fundamental for understanding how the organizer robustly deals with tissue growth.

Altogether, we have shown that a modeling tool based upon a mechanical approach to the dynamics of tissue growth contributes to the quantitative and predictive understanding of the morphogenetic mechanisms that govern the evolution of the wing imaginal discs of Drosophila. This tool can feedback to in vivo experiments of the DV organizer in order to test our predictions and also be potentially implemented in other growing tissues where cell packing dynamics and biomechanical interactions are key elements.

## Methods

### Dynamical Vertex Model

The vertex model assumes that each cell can be represented by a discrete set of points: the apical vertices that define its characteristic polygonal-like morphology. Each vertex evolves independently and off-lattice driven by the biomechanical properties of the surrounding cells. It is assumed that the dynamics is purely relaxational, in the sense that it derives from the minimization of a (time-dependent) energy functional. Extending previous formulations, [50], the energetic contribution of each vertex 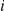 takes the form:
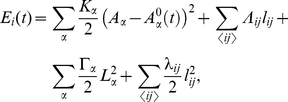
(2)


where the sums indexed by 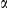 and 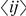 run respectively over the cells and vertices 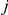 sharing the vertex 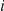. The first three terms on the right hand side have been previously proposed [50]. The first term accounts for the elastic energy of cells, 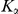 being proportional to the Young modulus, due to the difference between the actual cell area 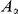 and the one that would have due to the cytoskeleton structure in the absence of the stresses associated to adhesion and cortical tension, 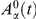. Note that the time-dependence of 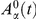 contains the information on the cell cycle (see below) and is what drives continuously the system out of equilibrium. The second term in Eq. (2) stands for the line tension, 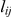 being the length of the edge connecting neighboring vertices 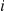 and 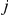, and includes contributions from cell-cell affinities (the action of molecules regulating the adhesion between cells, e.g. Armadillo) and also cortical tension, with the parameter 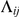 weighting these interactions. Finally, the last two terms model further contributions from the mechanical tension associated to the contraction of the actomyosin cortical ring. In this regard we distinguish two contributions. First, a term proportional to the squared cell perimeter, 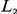, that takes into account a global contractility effect. On top of that, we consider a local contribution that accounts for possible inhomogeneities of the contractile tension due to the accumulation of actin-myosin in specific regions of the ring as experimentally reported [28], [29]. This last term is proportional to the squared edge length and herein will mimic the mechanical role played by the actin cable at the DV boundary.

By neglecting inertial effects with respect to dissipation, we are led to consider an overdamped dynamics with a characteristic kinetic coefficient 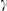. Then, the equation of motion for vertex 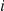 at position 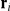 driven by the force 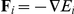 may be written as,
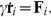
(3)


The characteristic relaxation time is assumed to be much smaller than the average cell cycle duration, implying that the vertex configuration adapts almost immediately to the (time-dependent) local minimum of the energy. Notice that our scheme differs from the quasistatic approach used in previous studies where concurrent growth of cells is neglected [39], [50]. Here we do not disregard the duration of the growing phase of the cell cycle. This makes our vertex model fully dynamical and more realistic even if 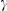 is very small. We also note that a relaxational approach does not imply that cells behave as merely passive elements. For example, the cellular growth certainly involves an active cytoskeleton remodeling. However, the active contributions are either time independent or assumed to adapt sufficiently fast with respect to the time scale of the relaxation dynamics so that they do not need to be treated explicitly.

In order to prescribe a biologically realistic evolution of the network topology (i.e. network of vertices connections) it is also necessary to include processes that may alter the cell neighbors environment, allowing for tissue plasticity: T1 recombination processes and T2 apoptosis/extrusion processes (see [51], [60]). T1/T2 processes are implemented in the in silico experiments when 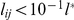, where 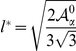 is a characteristic edge size defined as the length of a regular hexagonal cell with area 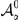, the minimum prescribed area of a cell (see below). Finally, we set the external boundary of the tissue to be free to move with the same dynamics but with a larger positive line tension to keep the tissue sufficiently compact, close to a circular shape.

### Cell Cycle, Cell Growth, and Cell Division

The cell cycle duration is not deterministic but stochastic and depends on cell-autonomous processes and on the mechanical interactions with the cell local environment. Both are considered in our approach. Moreover, during the course of the cell cycle two distinct growth phases can be distinguished. On the one hand, there is a latent phase up to the middle of the cell cycle during which the cell does not grow. On the other hand, during the rest of the cell cycle, the cell grows such that the apical cell area increases in an approximately linear manner [52], (Bellaiche Y (2008) Private communication). We account for these observations as follows.

Previous works have considered an internal clock, yet decoupled from the actual growth dynamics of the cell and its environment [52]. Herein, we first define an internal “clock” for each cell characterized by a temporal variable, 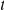, that measures the time elapsed since the beginning of the cell cycle. In addition we define the variable 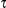,
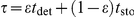



such that 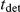 is a deterministic time scale that accounts for a mean cell cycle duration in the absence of mechanical stress due to the cell local environment and 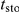 is a random variable that accounts for the variability of cell cycle duration and that is assumed to follow an exponential distribution:
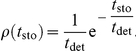



The parameter 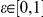 controls the dispersion of the cell cycle duration, so that, with the above definitions, the average and standard deviations of 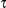 are given respectively by,
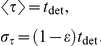



This approach for describing the cell cycle duration has been similarly hypothesized by other researchers and experimentally tested [61], [62], and reproduces the cell-age distribution such that, on average, the number of cells at the beginning of the cell cycle doubles that at the end of it following an exponential decay. Accordingly, here we choose 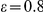. We then set the duration of the latent phase to be 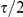.

As for the growing phase, we consider that the speed at which the apical area changes is the same for all cells such that,
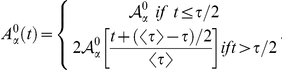



Thus, the time it takes for a given cell to double its preferred area is 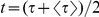 and its mean reads 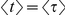. Yet, the *actual* duration of the cell cycle that leads to the division event is prescribed to take place when the *actual* cell area, 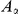, reaches a certain threshold. Here we choose this threshold to be 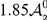 (Bellaiche Y (2008) Private communication). The latter enforces the duration of the cell cycle to depend on the intra- and inter-cellular mechanical interactions. At completion of the cell cycle the cell is assumed to divide instantaneously and the cellular clocks of both daughter cells are reset. In our simulations the phase of the cell cycle in the initial configuration is randomly chosen for every cell.

With respect to the cleavage direction, we implement the Hertwig rule that fixes a correlation between the longest axis of a cell and its division direction (transversely to the former) [63]. While there are several exceptions to this rule, e.g. Zebra fish gastrulation [40], it is highly conserved among cell types and organisms. In particular, this rule holds for the epithelial cells of the wing imaginal disc of Drosophila; yet, a dispersion that can be as large as 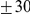 degrees has been registered (Bellaiche Y (2008) Private communication), [64]. Recent studies suggest that this correlation persists in the wing during all its developmental stages [45], [64]. Moreover, the influence of cell geometry on the positioning of the division plane has been thoroughly explored recently in other cell types (sea urchin eggs) [65]. This research has confirmed anew that the cleavage direction is set perpendicular to the longest axis of symmetry.

In our in silico experiments, as a novelty, in order to determine the longest axis at division time, we evaluate the inertia tensor of the cell with respect to its center of mass assuming that a proper representation of the former is a polygonal set of rods, i.e. the cell edges. Upon diagonalization of the inertia tensor we obtain the principal inertia axes and subsequently the longest axis of the cell (orthogonal to the direction along the largest principal inertia axis). Once the cleavage direction has been specified by these means we randomly (bounded Gaussian) implement a perturbation that may deviate the division axis up to 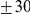 degrees as aforementioned. This finally defines the two new vertices and consequently the new edge.

### Cell Cycle Duration Differences: A Geometrical Argument

During development, Notch activity eventually controls cell proliferation of cells at the organizer by arresting the G1-S cell cycle progression [66], [67]. In fact, by late third instar the DV organizer and neighboring cells clearly define the so-called Zone of Non-proliferating Cells (ZNC) [66]. Thus, one may expect differences in the cell cycle duration of the organizer cells with respect to those at the compartments bulk [15], [55]. While a precise quantification of this effect with respect to the DV organizer within the temporal window of our interest (see below) is missing, a simple, yet elucidating, geometrical argument allows us to estimate and predict those differences as follows. The organizer grows in one dimension since its thickness (width) remains constant, as opposed to the whole disc that grows roughly isotropically in two dimensions. Let us then suppose a growing disc. The area of such disc is then 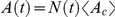 where 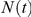 and 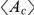 stand respectively for the number of cells and the average cell area. If 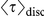 is the average cell doubling time, 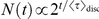, then 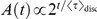 and consequently the disc radius grows as 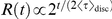. Therefore, if we want to make compatible the growth of the organizer within the disc we conclude that 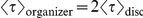. That is, the average cell cycle duration of the cells growing in one dimension must be twice the average cell cycle duration of the cells growing in two dimensions. This geometrical argument suggest that, before the ZNC becomes specified, Notch activity at the organizer contributes by approximately doubling the cell cycle duration with respect to that at the bulk of the compartments and helps to maintain the straightness and the width of the growing DV organizer.

### Dimensionless Parameter Values, Initial and Boundary Conditions

By choosing the following characteristic scales of length, 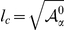, and time, 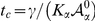, Eq. (2) can be written in dimensionless form by defining the constants 
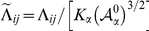
, 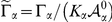, and 
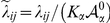
. The assumption of fast mechanical relaxation (driven by the energy functional) compared to cell growth in our dynamical model thus implies 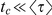.

Whereas in the AP case a size difference between cells at the boundary and cells at the compartments has been reported [39], so far there is no quantitative evidence that the same happens for the DV boundary. Consequently, herein we assume that for all cells, i.e. for all 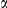, 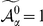 (in dimensionless units, 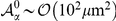).

As shown in the Results section, we make use of different in silico experiments that we compare with in vivo experimental data for setting a meaningful value of the parameters: the histograms of the number of cell sides [50], the cell area distribution [50], the cell cleavage direction statistics [28], [41], and the dynamics of ablation experiments [50]. An initial guess of some parameters was taken from [50] and [39]. Still, the particularities of our problem from the biological and technical point of view leads to different, yet close, parameter values. In addition, the average duration of the cell cycle is also available, 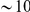 hours (we use this value for cells at the bulk of the compartments and ∼20 hours for organizer cells.), which allows us to compute the area growing speed [68]. In order to test the effect of the mechanical interactions between cells for determining the *actual* duration of the cell cycle, in our simulations we also compute the latter and found 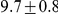 hours for cells at the bulk and 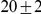 hours for cells at the organizer (see Fig. S1). Moreover, the ablation experiments provide a temporal scale for the mechanical relaxation (see Results section): 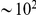 seconds [50]. By comparing these scales, we can state that the mechanical relaxation is approximately a hundred-fold faster than the growing speed. These scales can also be compared with the typical scale of gene expression processes. While the latter greatly depends on the problem under consideration, it is in any case faster than the cell cycle duration. For example, the timescale for the transcription and translation of Wingless is of the order of 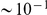 hours [69]. Consequently, with respect to the growing time scale of the cell, the gene expression dynamics can be adiabatically eliminated and remains stationary.

Our initial condition assumes a set of 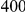 regular hexagonal cells with a two-cells-wide stripe (the fence) specified as the DV organizer. We stress that this population corresponds to the signaling center from which Wingless is released. Yet, we do not consider the diffusion process but just its consequences with respect to the maintenance of the cellular character. In that regard, following a division event the “genetic” characteristics of the cell, in terms of its mechanical parameters, are inherited by its daughter depending on the cellular environment (see below). The values of the mechanical parameters are chosen as follows. The global contractility parameter is the same for all cells in the primordium: 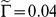. However, the line tension (“affinity”) between cells depends on the cell type [31], [37]: 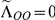, 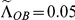, and 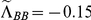. Where the subscripts 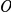 and 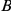 denote organizer cells and cells at the compartment bulk respectively.

Thus, in a compartment, the affinity between cells at the bulk is maximal, and the less favorable situation in terms of the line tension corresponds to the mixing between organizer cells and those of the compartment bulk. This in turn favors cell segregation. In addition, to account for the actin cable effects we distinctly prescribe: 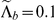 and 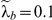 for edges defining the DV boundary, i.e. edges shared by organizer cells of different compartments (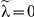 for all other edges in the in silico disc).

As for the boundary condition, most authors that have implemented the vertex model have dealt with it using periodic boundary conditions. That simulation scheme implies that an overall tissue growth rate must be imposed ad hoc. Herein, we use free boundary conditions. Yet, the line tension of the in silico disc border, i.e. edges facing the exterior, is set to 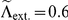. The latter ensures that the tissue adopts a compact, roughly circular, geometry as seen in the wing pouch. Moreover, the main effect of the external line tension is to introduce an overall external pressure to the tissue, an effect that will always be present although one cannot easily quantify due to lack of experimental information. Yet, it is possible to deal with it effectively by including such external line tension.

### Parameters Variation: Robustness Analysis

The aforementioned values of the parameters for the wild-type provides the best results in terms of the comparison with the available experimental data and the robustness to mechanical perturbations (see Results section). We have also tested the robustness of our model with respect to parameters variation. That analysis is performed as follows. Taking as a reference 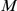 wild-type parameters, 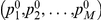, we generate for each parameter, 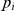, a new value at random (uniform distribution) that allows a variation, 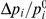, up to 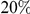 (up or down). We note that in our case, we exclude from this analysis the value of the line tension at the tissue periphery and the null value of 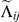 for edges of the organizer cells that do not define the DV boundary, i.e. 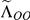. That is, we allow 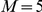 parameters to vary. Each parameter set 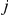 is then characterized by the vector 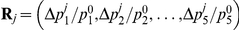. Consequently, the null vector 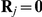 corresponds to the wild-type situation.

We notice that we check that the random sampling comprehensively explores the parameter space and that the obtained sets are scattered enough such that given any two set of parameters 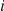 and 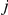 then 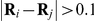. In order to quantify the amount of variability for each parameter set, we compute the Euclidean distance to the wild-type condition, that is, the norm of the parameter vector 
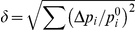
. Consequently, in our analysis the maximum variation with respect to the wildtype condition is 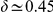 (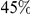).

We then run simulations with the new parameters set and check whether or not the organizer breaks. In total we performed simulations for 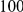 different parameter sets. In addition, in order to analyze the effect of the external line tension, we perform simulations where we just vary this parameter up to 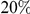.

### Temporal Window of Interest: Maintenance of the Cellular Character

The dynamics of the DV organizer displays several, well differentiated, stages during development. Covering all of them is out of the scope of this study. We now briefly review some of these processes in order to better specify the temporal window we aim to describe. The DV boundary appears in middle stages of larval development [13]. In the middle of the second instar Apterous expression patterns the dorsal region of the wing disc [70]. Apterous first drives the Notch activation onset by promoting that dorsal/ventral Notch receptors get signaled by ventral/dorsal Delta/Serrate ligands [36]. This causes a two-three cells Notch activation stripe due to the receptor-ligand dynamics between opposing compartments. Herein we disregard this initial dynamics for activating Notch. Such incipient Notch activation is further amplified and stabilized as Wingless and Cut become expressed at boundary cells. Remarkably, Cut activity, which is evident by mid-third instar [36], makes cells at the DV organizer refractory to Wingless signals that represses Notch activity via Dishevelled [27], [71]. The latter removes ligands at the DV organizer cells and promotes polarized signaling: in order to maintain Notch activity DV organizer cells are forced to recruit ligands from cells adjacent to, but outside, the organizer [27]. In other words, at this stage, due to Cut activity, Notch activity is stabilized and sustained by cells of the compartment bulk adjacent to the DV organizer and not by ligands of opposite compartments as in the previous stage. We notice that in the past it was postulated that the stripe of early arising cut-expressing cells might be the barrier that separates dorsal and ventral compartments [72]. Moreover, the actomyosin cable that is regulated by Notch activity is evident at the beginning of the third instar and persists past the middle of the third instar [28], [29]. Our modeling assumes this situation, i.e. early-mid third instar, as the initial timepoint and follows the dynamics during the next 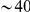 hours. Later, as development progresses, around 48 hours after the beginning of third instar, the boundary cannot be identified by F-actin staining, and two new cables start to develop at flanking cells [28]. In this study we do not consider these phenomena either.

As mentioned above, our model accounts for two cells types with distinct mechanical and dynamical characteristics: cells at the compartment bulk, *C*, and cells at the DV organizer, *O*. According to the previous discussion, an *O* cell maintains its character as long as is in contact with a *C* cell. Namely, signaling from ligands at the bulk is necessary and sufficient for sustaining Notch activity at the organizer. While Notch-ligand signaling is supposed to happen between adjacent cells, recent studies have unveiled long range interactions. Thus, actin-based filipodia confer robustness to the Notch-Delta signaling mechanism and extend this interaction to cells that are not nearest neighbors [59]. Everything considered, in our model the *O* character of a cell is maintained if one, or more, nearest neighbor or next-nearest neighbor has a *C* character. Otherwise the *O* cell becomes a *C* cell: Notch activity is lost. In addition, we do not consider the reverse step: a *C* cell cannot turn into a *O* cell. Notice that the latter requires the initial onset of Notch activity established at previous developmental stages for eliciting Cut expression since Wingless signaling from *O* cells inhibits Notch activation in *C* cells.

### Computer Implementation of In Silico Experiments

We develop our own code according to the prescriptions mentioned above. The algorithm for integrating Eq. 3 is a standard time-explicit FTCS (Forward Time Centered Space). The code makes use of the parallel CUDA technology as we run our simulations in the computer GPU (NVidia GeForce GTX 295).

## Supporting Information

Figure S1Position of the division events and actual duration of the cell cycle. When a cell divides, the position of the mother cell is marked by a circle just before the cleavage event. The color code indicates the actual duration of the cell cycle. In average, the actual duration of the cell cycle is 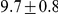 hours for cells at the compartment bulk and 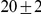 hours for cells at the organizer.(PDF)

Figure S2Effect of the external line tension. We perform simulations varying the external line tension up to 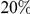. If that parameter is decreased by a 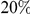 with respect the wild-type situation, the organizer develops robustly (left). When the external line tension is increased by a 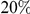, then the organizer threaten to break at some locations (right). Still, in all cases the dynamics is similar to the wild type and the organizer keep the two compartments segregated.(PDF)

Video S1 Dynamics of DV organizer (red) in an in silico wild-type wing imaginal disc.
(AVI)

Video S2Correlations in the cell cycle phase. Here all cells have a bulk character and the initial phase of each cell is taken at random. The color code indicates the progression of the cell cycle: red
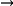
yellow
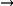
green
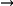
blue. A cell and its progeny maintain the coherence and synchronization of the cell cycle phases during a temporal window. Yet, in the long term, the stochasticity in the duration of the cell cycle induces asynchrony.(AVI)

Video S3 In silico laser ablation of the edge of a cell with a bulk character. Left: cells with an ablated edge appear in red. Right: separation of the vertexes that correspond to the ablated edge as a function of time.
(AVI)

Video S4 A clone of rapid duplicating cells (blue) exerts a differential pressure over the DV organizer (red) that bends. Still, under wild-type conditions, the organizer remains intact and restricts the cellular lineages.
(AVI)

Video S5 An ectopic organizer (red) evolves minimizing the surface energy. The organizer is initially set with an eight-shaped conformation and ends with a circular one.
(AVI)

Video S6 The removal of the organizer produces cell mix. In the absence of an organizer, cells from opposite compartments do not respect the DV boundary and start to intermingle.
(AVI)

Video S7 Differential affinities are required for a robust development of the organizer. If all cells have the same affinity then the DV organizer easily breaks.
(AVI)

Video S8 The actomyosin cable removal produces a DV organizer that lacks robustness. While in this video there is not cell intermingling the organizer is only one cell wide at different locations and threatens to break.
(AVI)

Video S9 The Hertwig rule is required for keeping cell segregation. If no rule for cell cleavage is prescribed the DV organizer breaks. Here the cleavage direction is set at random.
(AVI)

Video S10 Effect of the cell cycle duration. If there is not a differential cell cycle duration of the cells at the DV organizer with respect to cells at the bulk, the organizer becomes wider and the boundary wiggly.
(AVI)
