# Supplementary figures and images for: Dynamics and Mechanical Stability of the Developing Dorsoventral Organizer of the Wing Imaginal Disc
Source: PLoS Comput Biol. 2011 Sep 29;7(9):e1002153. doi: 10.1371/journal.pcbi.1002153 (PMC3182857; doi:10.1371/journal.pcbi.1002153)

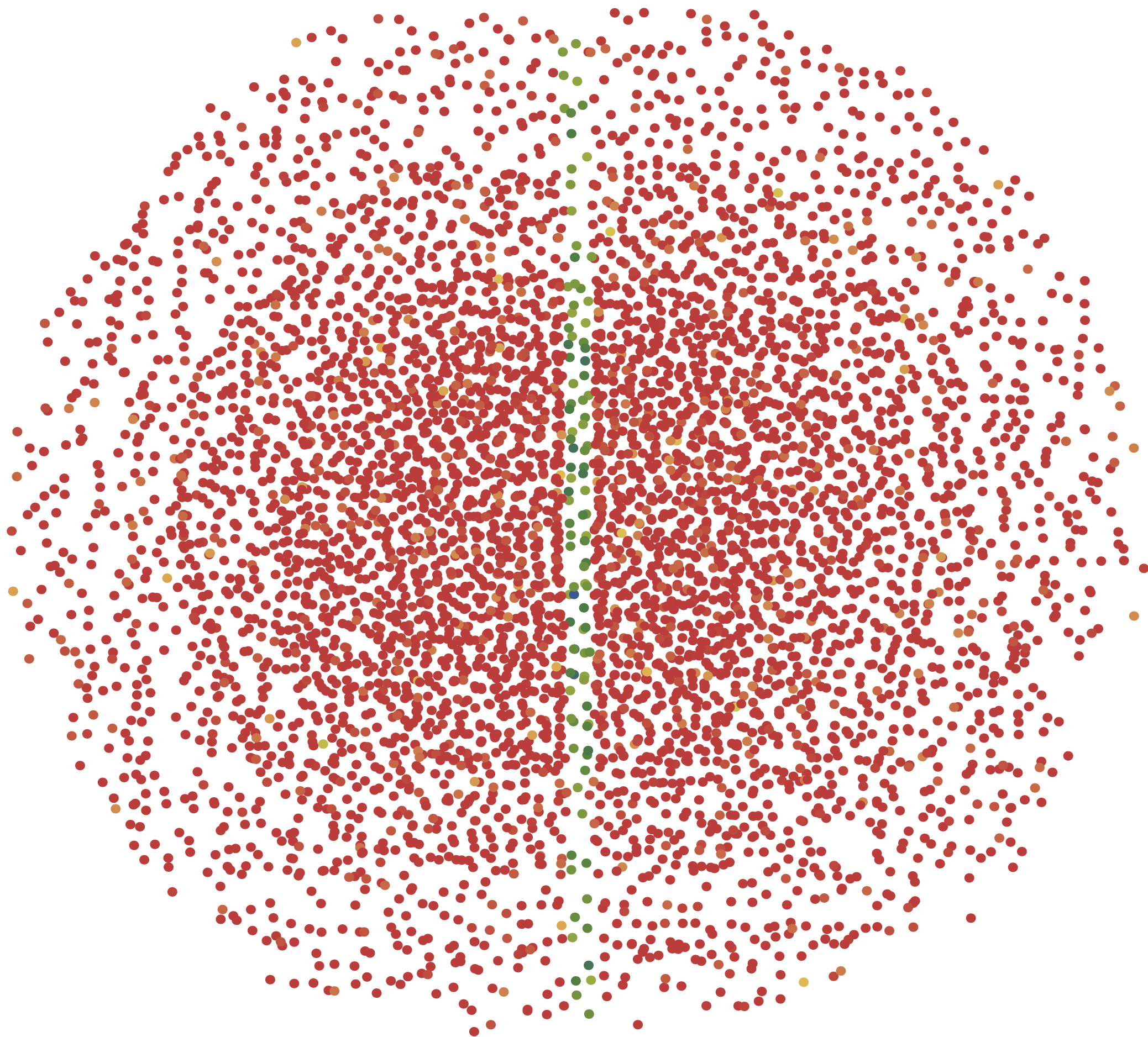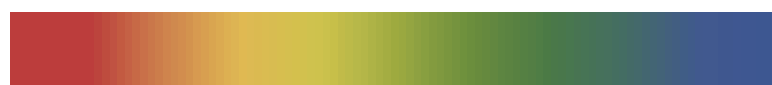

$\sim 8.3h$

$\sim 27.5h$

Supplement: Figure S1 — Position of the division events and actual duration of the cell cycle. When a cell divides, the position of the mother cell is marked by a circle just before the cleavage event. The color code indicates the actual duration of the cell cycle. In average, the actual duration of the cell cycle is hours for cells at the compartment bulk and hours for cells at the organizer. (PDF) [file pcbi.1002153.s001.pdf]

$$\tilde{\Lambda}_{ext} = 0.48$$

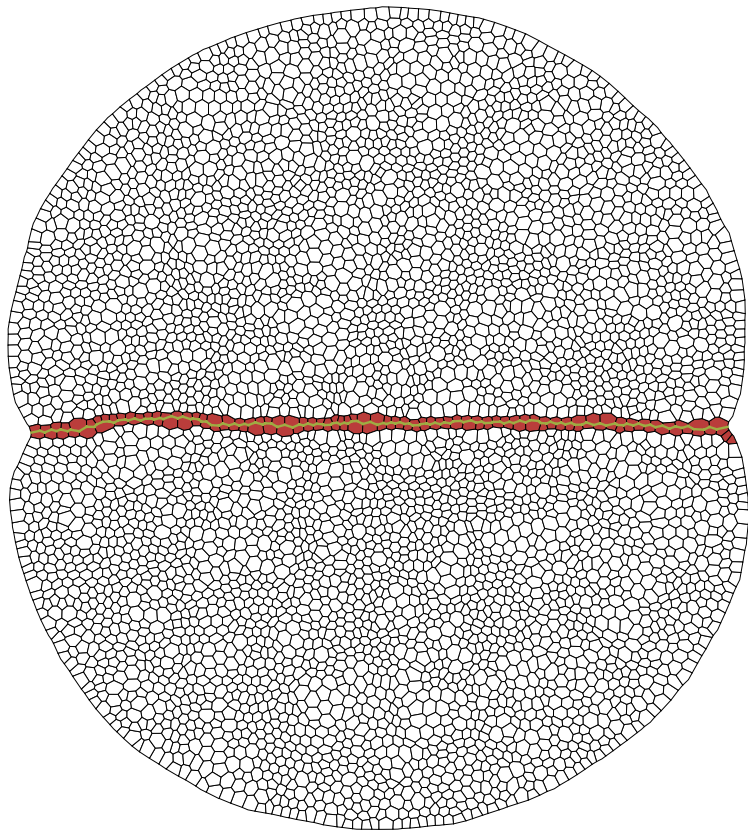

$$\tilde{\Lambda}_{ext} = 0.72$$

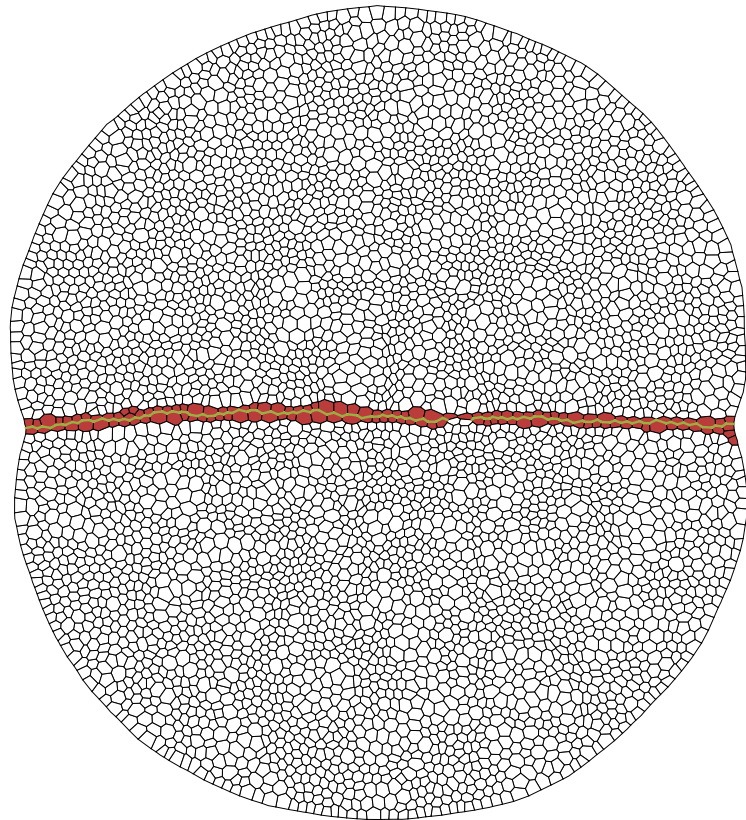

Supplement: Figure S2 — Effect of the external line tension. We perform simulations varying the external line tension up to . If that parameter is decreased by a with respect the wild-type situation, the organizer develops robustly (left). When the external line tension is increased by a , then the organizer threaten to break at some locations (right). Still, in all cases the dynamics is similar to the wild type and the organizer keep the two compartments segregated. (PDF) [file pcbi.1002153.s002.pdf]
